# The mitochondrial protein Sod2 is important for the migration, maintenance, and fitness of germ cells

**DOI:** 10.3389/fcell.2023.1250643

**Published:** 2023-10-26

**Authors:** Katsiaryna Tarbashevich, Laura Ermlich, Julian Wegner, Jana Pfeiffer, Erez Raz

**Affiliations:** ^1^ Institute of Cell Biology, Center for Molecular Biology of Inflammation (ZMBE), Muenster, Germany; ^2^ Max Planck Institute for Molecular Biomedicine, Münster, Germany

**Keywords:** SOD2, mitochondria, germ cells, zebrafish, cell competition, cell migration, PLLp, posterior lateral line primordium

## Abstract

To maintain a range of cellular functions and to ensure cell survival, cells must control their levels of reactive oxygen species (ROS). The main source of these molecules is the mitochondrial respiration machinery, and the first line of defense against these toxic substances is the mitochondrial enzyme superoxide dismutase 2 (Sod2). Thus, investigating early expression patterns and functions of this protein is critical for understanding how an organism develops ways to protect itself against ROS and enhance tissue fitness. Here, we report on expression pattern and function of zebrafish Sod2, focusing on the role of the protein in migration and maintenance of primordial germ cells during early embryonic development. We provide evidence that Sod2 is involved in purifying selection of vertebrate germ cells, which can contribute to the fitness of the organism in the following generations.

## Introduction

The control over the level of reactive oxygen species (ROS) in the cell is important for a range of physiological and pathological processes (reviewed in (Bowling et al., 2019; Lawlor et al., 2020; Sie et al., 2020; Shields et al., 2021)). A major source of these harmful ROS compounds is oxidative phosphorylation in the mitochondria, but mitochondria also have a way to handle them: ROS are neutralized by detoxifying enzymes such as superoxide dismutase 2 (SOD2), a mitochondrial enzyme that converts superoxide radicals into the less harmful compound hydrogen peroxide. Consequently, the function of SOD2 is critical for cell survival in *Drosophila* (Celotto et al., 2012) and for the immune response in zebrafish (Peterman et al., 2015). Mice that have a reduced SOD2 function are more likely to develop epilepsy (Liang and Patel, 2004), cardiomyopathy defects (Van Remmen et al., 2001), and oncological and aging-related phenotypes ((Justilien et al., 2007; Velarde et al., 2012; Weyemi et al., 2012); reviewed in (Lee et al., 2009)).

While SOD function has been shown to be important in different cell types, the role of the protein during the early development of vertebrate embryos is not well understood, in particular regarding its role in selecting for fitter germline cells that ensure the robustness of the organism across generations. So far, the process of “purifying selection” based on cell competition, namely, the selection for cells with optimal energy supply or metabolic status, has been described for somatic cells during early mouse embryogenesis ((Döhla et al., 2022; Lima et al., 2021)). In the mouse, natural selection of male germ cells was reported to be based on differentiation efficiency but the impact of metabolic parameters on the process was not analyzed ((Nguyen and Laird, 2021; Nguyen et al., 2020)). Thus, not all aspects of competition among germ cells have been studied. To explore these issues, we employed the zebrafish model, benefiting from the extrauterine development of the embryo and the available genetic and reverse genetic tools. We examined the role of Sod2 in early embryogenesis with respect to cell maintenance as well as collective and single cell migration, focusing on its function in germ cells. We show that Sod2 is expressed in germ cells and is important for their development and provide evidence that Sod2 protein is involved in the selection for fitter germ cells, thereby contributing to the fitness of the organism across generations.

## Materials and methods

### Fish lines and husbandry

The following zebrafish (*Danio rerio*) lines were employed: wild-type of the AB background, *Tg(kop:EGFP-F’nos3′UTR)* (Blaser et al., 2005)*, Tg(kop:mcherry-F-nos3′UTR)* (Tarbashevich et al., 2015)*, Tg(gsc:GFP)* (Doitsidou et al., 2002)*, Tg (cldnB:lynEGFP)* ^*+/+*^ (Haas and Gilmour, 2006)*, mu13* (MZ*sod2* ^*STOP31*^) *Tg(kop:EGFP-F’nos3′UTR), mu14* (MZ*sod2* ^*STOP19*^) *Tg (cldnB:lynEGFP)* ^*+/+*^.

WT and manipulated embryos were collected, kept in 0.3× Danieau’s solution [17.4 mM NaCl, 0.21 mM KCl, 0.12 mM MgSO_4_·7H_2_O, 0.18 mM Ca(NO_3_)_2_, and 1.5 mM Hepes (pH 7.6)], and raised at 28°C. The general zebrafish maintenance was performed in compliance with the German, North-Rhine-Westphalia state law, following the regulations of the Landesamt für Natur, Umwelt und Verbraucherschutz Nordrhein-Westfalen and was supervised by the veterinarian office of the city of Muenster.

### Microinjections into the zebrafish embryos

For all experiments except for the one presented in Figure 7, embryos were microinjected into the yolk with 2 nL of the injection mixture. The following capped sense mRNAs synthesized using the mMESSAGE mMACHINE (Thermo Fisher Scientific): GrpEL-EGFP (C131.GrpEL.EGFP.nos) (used as mitochondria marker for co-localization studies), Sod2-mCherry (C247.Sod2-mCherry-nos) (used to determine intracellular protein localization), Sod2 (C382.Sod2.nos, E046.Sod2ORF.sod2 3′UTR) (both used for rescue experiments), mCherry-H2B (B325. mCherry.H2B.globinUTR) (labels nuclei of all cells in the embryo, used for the correction for the gastrulation movement in the cell migration analysis), Mito-mCherry (C433.mCherryHA-Bcl2TM.nos3′UTR) (used for the fluorescence-based analysis of the mitochondria numbers in the PGCs).The mRNAs containing *nos* 3′UTR are cleared from somatic cells via miRNA430-mediated degradation and are, thus, predominantly expressed in germ cells (Kedde et al., 2007).

Translation-blocking morpholinos (MO, Gene tools) utilized for this work were: CntrMO (CCT​CTT​ACC​TCA​GTT​ACA​ATT​TAT​A), Sod2MO (CAT​GCT​CTA​GTC​CGT​CAC​ACA​GTG​A). Both morpholinos were injected in 0.5 µM concentration.

For the experiment described in Figure 7, 8-cell-stage embryos were microinjected into one of the middle blastomeres with 1 nL injection mix containing Cas9 nuclease (0.5 μg/μL, Integrated DNA technologies (IDT), cat. Nr. 1081059), guide crRNA *egfp* (18 ng/μL, 5′-AAG​GGC​GAG​GAG​CTG​TTC​AC-3′), guide crRNA *sod2* (18 ng/μL, 5′-ACA​AAT​CTG​TCA​CCC​AAT​GGC​GG-3′), tracrRNA (33.5 ng/μL, cat. Nr. 1072534) according to the manufacturer’s recommendations (IDT).

### Transplantation experiments

For the posterior lateral line primordium (pLLP) transplantation experiments, wild-type and MZ*sod2* ^*STOP31*^ embryos were injected with 500 pg of dextran conjugates (Invitrogen™, Dextran, Alexa Fluor™ 568; 10,000 MW, Anionic, Fixable, Catalogue number: D22912 or Alexa Fluor™ 680; 10,000 MW, Anionic, Fixable, Catalogue number: D34680) of distinct colors and used as donors. Uninjected wild-type embryos carrying a *cldnB:lynEGFP* transgene served as hosts.

Equal numbers of both types of donor embryos were grown to 4 h post fertilization (hpf), before removing the yolks. The cell caps were combined in a tube and carefully dissociated by tapping, until a homogeneous cell mixture was obtained. This cell mixture was transplanted into shield stage host embryos. At 31 hpf, dextran-labeled donor cells were distributed throughout the host embryos. Transplanted embryos were screened for cases in which both wild-type and MZ*sod2* ^*STOP19*^ donor cells had integrated into the host pLLP. The primordium was then imaged with Z-steps of 20 µm.

For the PGC transplantations, wild-type (WT) (*Tg(kop:EGFP-F’nos3′UTR)* ^*+/+*^) or *sod2* knockout (KO) (MZ*sod2* ^*STOP31*^ *Tg*(*kop:EGFP-F’nos3′UTR*)^*+/+*^) germ cells from 4.7-5 hpf donor embryos expressing EGFP-F′ transgene and dominant marker *Tg*(*cry:DsRed*) (red eyes) were transplanted into 4 hpf hosts (*Tg(kop:mcherry-F-nos3′UTR)*) expressing mCherry-F′ transgene and dominant marker *Tg(cmlc:EGFP)* (green heart) or into “dark” MZ*sod2* ^*STOP31*^ embryos. Embryos with mosaic germ cell clusters were selected at 24 hpf and raised to adulthood.

### Immunohistochemistry

Whole-mount *in situ* hybridization using the DIG-labeled probe for the *sod2, egr2a*, *mab21l*, *en2a* and *foxa3d* was performed as previously described (Weidinger et al., 2002).

RNAscope was performed as described previously (Gross-Thebing et al., 2014) using the following probes: *sod2* (cat. Nr. 300031-C2/320269-C2), *vasa* (cat. Nr. 407271-C3), *cxcl12a* (cat. Nr. 406481), *noto* (cat. Nr. 4835511-C2), *gata1*(cat. Nr. 473371-C3), *neurog1* (cat. Nr. 505081-C2), *myoD* (cat. Nr. 402461-C2), *pax8* (cat. Nr. 494721-C2), *pcdh8* (cat. Nr. 494741-C3).

Fluorescence whole-mount immunostaining of the telomere repeats was performed using TelC-Cy3 (cat. Nr. TP-007) and TelC-Cy5 (cat. Nr. TP-009) according to the manufacturer’s instructions (PNA Biotech).

### Image acquisition and microscopy

For live imaging, embryos were dechorionated, and those older than 20 hpf were anesthetized using tricaine (concentration, A5040, Sigma-Aldrich) in 0.3× Danieau’s solution, mounted in agarose-coated ramps covered with Danieau’s solution and manually oriented. For experiments aimed at determining migration parameters, samples were incubated at 28°C on a heated stage (PeCon, TempController 2000-2).

Spinning disk confocal microscopy was performed using Carl Zeiss Axio Imager Z1 and M1 microscopes equipped with a Yokogawa CSUX1FW-06P-01 spinning disk units. Imaging was performed using a Hamamatsu ORCA-Flash4.0 LT C11440 camera and Visitron Systems acquisition software (VisiView). Imaging of RNAscope samples (×10 objective) was conducted by acquiring 400-μm *z*-stacks (80 Z planes, 5 μm apart). For the analysis of PGC speed, ×10 objective multi-stage time-lapses were acquired for 2 h with 2-min acquisition intervals. For the PGC blebbing analysis, ×63 objective high-magnification movies of individual cells were acquired with 5-s intervals for 5 min. For the neomycin treatment, dechorionated embryos were incubated in a 400 µM neomycin (Sigma, cat. Nr. N1142) solution in Danieau’s buffer from 3 hpf until the end of the experiment. For the control treatments, the same amount (in µl) of DMSO (Sigma, cat. Nr. D8418) was used.

For the analysis of the pLLP migration, time-lapse movies were acquired at a rate of one frame every 30 min between 24 hpf 31 hpf. The samples were imaged using a1×0 objective to obtain150-µm Z-stacks of 10 µm optical slices.

High-magnification images for determining cell positioning and volume of the pLLP (40x) were performed by acquiring 2-μm optical slices. Analysis of the pLLP volumes was performed using the surface function of the Imaris software (Bitplane, version 9.5.1.).

Confocal laser scanning image acquisition was performed using LSM710 (Zeiss) upright microscope and ZEN software (Zeiss). If not stated otherwise, imaging was performed using a ×63 water-dipping objective with 2-μm optical slices.

### Determination of the embryo size

Determination of zebrafish embryo size at the 5-somite stage was performed using the Fiji distribution function of ImageJ (NIH). First, the nuclei signal (Hoechst) of RNAscope image data, was projected along the *Z*-axis (400 µm in total), performing a maximum intensity projection. Rolling ball background-subtraction (radius = 50 pixels) was subsequently applied to the projected image. Next, a binary image was created by thresholding the image using the Otsu algorithm and adjusting the threshold such that the entire embryo was included. The “Fill holes” function was used to fill the area between the nuclei that were previously not segmented. The area of the obtained embryo segmentations was measured (for wild-type and MZ*sod* ^*STOP31*^ embryos).

### Analysis of cell migration

Analysis of the PGC migration speed was performed by tracking the germ cells using Imaris software (Bitplane, version 9.5.1.), with the correction of the gastrulation movement of the surrounding somatic cells. Analysis of PGC blebbing activity was performed manually (bleb count) using Fiji software. To determine the portion of blebs generated by PGCs at the back of a cell relative to the total number of blebs, the cells were divided into front and back halves and the total number of blebs, as well as the number of blebs at the back was counted for 5-min time-lapse movies.

For the analysis of the PGC cluster length, the value obtained for a cluster on one side of an embryo was normalized to the length of the yolk extension of the given embryo. Analysis was performed using Fiji software.

Analysis of the pLLP migration speed and distance were performed using Fiji software. The image orientation was adjusted, such that the lateral line was perfectly horizontal, with the primordium traveling to the right. The anterior point of the yolk extension was used as the point from which the distance to the tip of the traveling primordium was measured. The obtained values were normalized to the average wild-type pLLP speed/distance of travel for the given experiment.

### qPCR

qPCR was performed as described in Cawthon (2009). Telomere primers: 5′ CGG​TTT​GTT​TGG​GTT​TGG​GTT​TGG​GTT​TGG​GTT​TGG​GTT-3′ and 5′- GGC​TTG​CCT​TAC​CCT​TAC​CCT​TAC​CCT​TAC​CCT​TAC​CCT-3’. The house keeping gene *hmg (high mobility group)* was used as a reference RNA amplified with primers 5′-GTG​GAA​GAC​CCC​GAA​AAC​A-3′ and 5′- TTC​CTC​CTC​TTC​TTC​CTC​CTC​G-3’. Additional normalization was performed to the values of the WT group.

### Western blotting analysis

Western blotting analysis was performed on protein extracts from wild-type and MZ*sod2* ^*STOP*^ embryos of 24hpf. The following primary antibodies were used: anti-Sod2 antibody (Proteintech, #24127-1-AP, in 1:3000 dilution) and a pan-Actin antibody (Thermofisher, #MA1-744, in 1:3000 dilution). For detection the following secondary antibodies were employed: goat-anti-mouse (Li-Cor, 926-68071, IRDye 680RD) and donkey-anti-rabbit (Li-Cor, 926-32213, IRDye 800RD) in 1:5000 dilution.

### ROS detection

For the detection of the level of the oxidative stress, 1hpf wild-type and MZ*sod2* ^*STOP*^ embryos were manually dechorionated and incubated for 3 h in 10 µM CellRox (ThermoFisher, C10422) in Danieau’s at 28°C followed by removal of the chemical and confocal imaging.

### Statistical analysis

Statistical analysis (one-way ANOVA or Student’s t-test) was performed using GraphPad Prism software (version 8). If not stated otherwise, all experiments were performed in three independent replicates. Where applicable, multiple comparisons were statistically analyzed using Dunn’s multiple comparison test.

## Results

### sod2 mRNA is enriched in migratory cell populations

**FIGURE 1:**
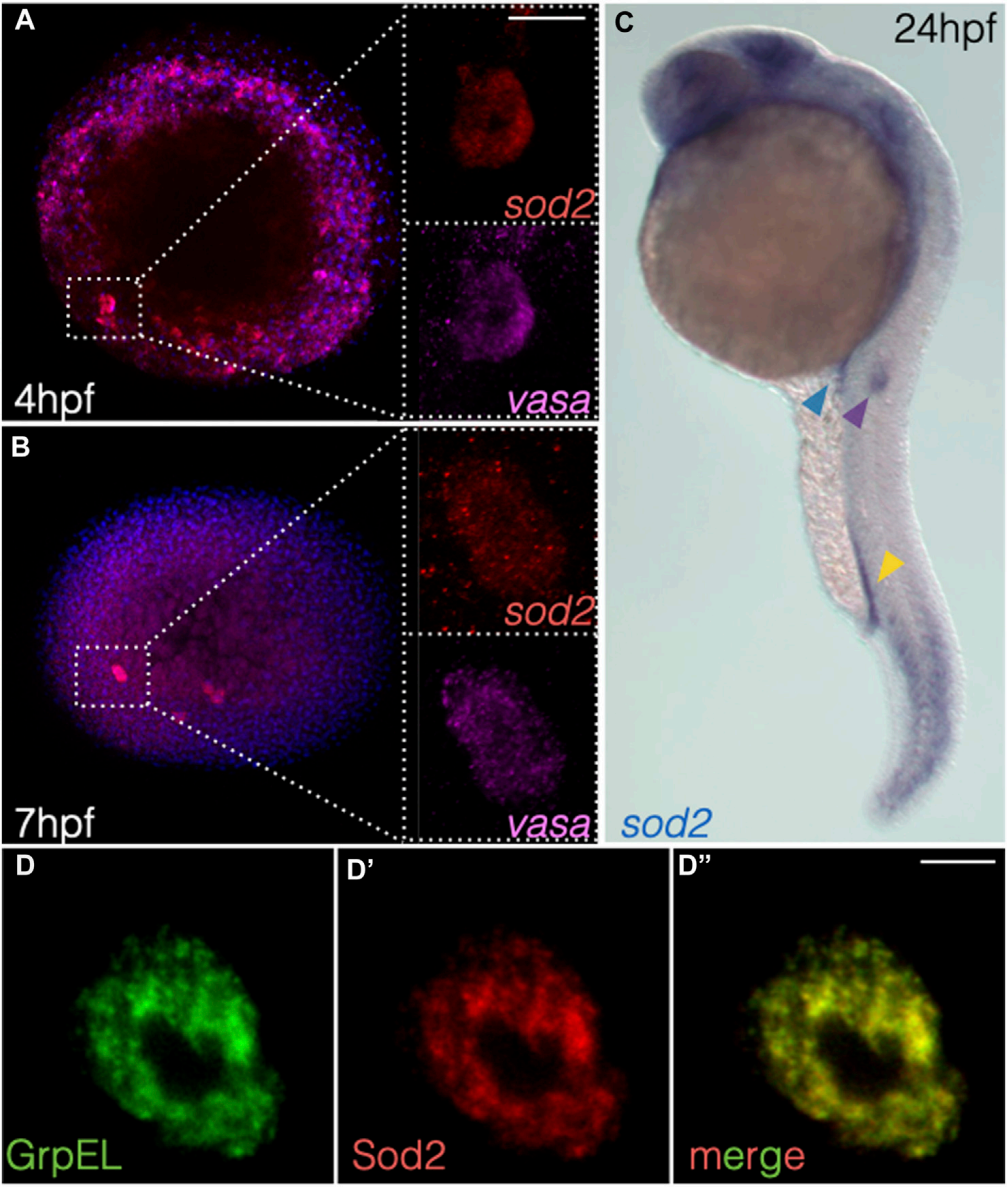
*sod2* RNA expression pattern and localization of the protein during early embryogenesis. **(A, B)** RNAscope-based localization of *sod2* mRNA and the germ cell marker *vasa* mRNA at **(A)** 4 hpf and **(B)** 7 hpf. **(C)** Whole-mount *in situ* hybridization image probing for *sod2* mRNA in 24 hpf embryos. Blue arrowhead points at the PGC cluster, yellow arrowhead at hematopoietic precursor cells, and purple arrowhead at the migrating pLLP. **(D–D’’)** Representative confocal images of Sod2-mCherry protein in germ cells relative to the mitochondrial marker (GrpEL-EGFP). Scale bar 10 µm.

Upon screening for RNA molecules enriched in zebrafish primordial germ cells (deep sequencing data published in (Hartwig et al., 2014; Paksa et al., 2016)), we identified *superoxide dismutase type 2* (*sod2*) (ZFIN:ZDB-GENE-030131-7742). During early stages of embryogenesis (4 and 7 h post fertilization (hpf)), we found that *sod2* mRNA was expressed globally, but enriched in primordial germ cells (PGCs) (Figures 1A,B, Supplementary Figure S1A). At later stages of embryogenesis, *sod2* expression was detected in several structures such as the caudal somites as well as in specific regions within the developing brain (Figure 1C, Supplementary Figure S1A and Supplementary Figure S2). Intriguingly, in addition to being expressed in the PGCs (blue arrowhead in Figure 1C, Supplementary Figure S1, Supplementary Figure S2), *sod2* RNA was strongly expressed within two other populations of migratory cells: caudal hematopoietic trunk cells (CHT) (yellow arrowhead in Figure 1C, Supplementary Figure S1A) and cells of the posterior lateral line primordium (pLLP) (purple arrowhead in Figure 1C, Supplementary Figure S1A and Supplementary Figure S2). Consistent with its function, the Sod2-mCherry fusion protein localized to the mitochondria of cells of early embryos (e.g., in germ cells expressing the mitochondrial protein GrpEL (Figure 1D-D’’)) (Srivastava et al., 2017).

### Generation of sod2 maternal zygotic mutant fish lines

**FIGURE 2:**
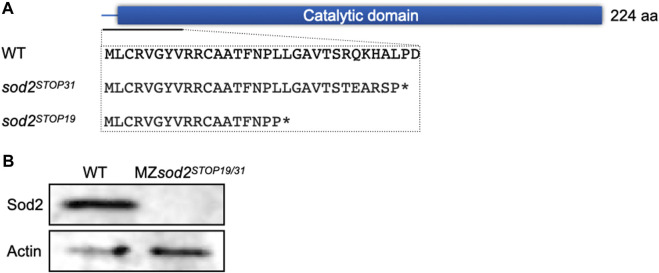
Generation of *sod2* maternal zygotic fish lines. **(A)** The schematic representation of the position of the two Cas9-induced mutations. The mutations resulted in stop codons at amino acid position 31 (*sod2* ^*STOP31*^) and 19 (*sod2* ^*STOP19*^) (marked by asterisks). **(B)** Western blotting analysis of the Sod2 protein expression in wild type and MZ*sod2* embryos.

To study the function of Sod2 during early zebrafish embryogenesis, we generated two knockout (KO) alleles employing CRISPR/Cas9 technology. Both lines carry mutations at the beginning of the catalytic domain of the wild-type (WT) protein, resulting in frameshifts and stop codons shortly following the mutation sites (Figure 2A). Thus, we considered the two mutated alleles to result in full loss of function of Sod2 (see below).

Since *sod2* transcripts (and presumably the protein) are maternally provided to the embryo, we aimed at generating adult mutant fish that were homozygous for both knockout alleles. In this way, one can obtain embryos that do not contain maternally-provided material that are also incapable of expressing functional RNA from their own genome.

**FIGURE 3:**
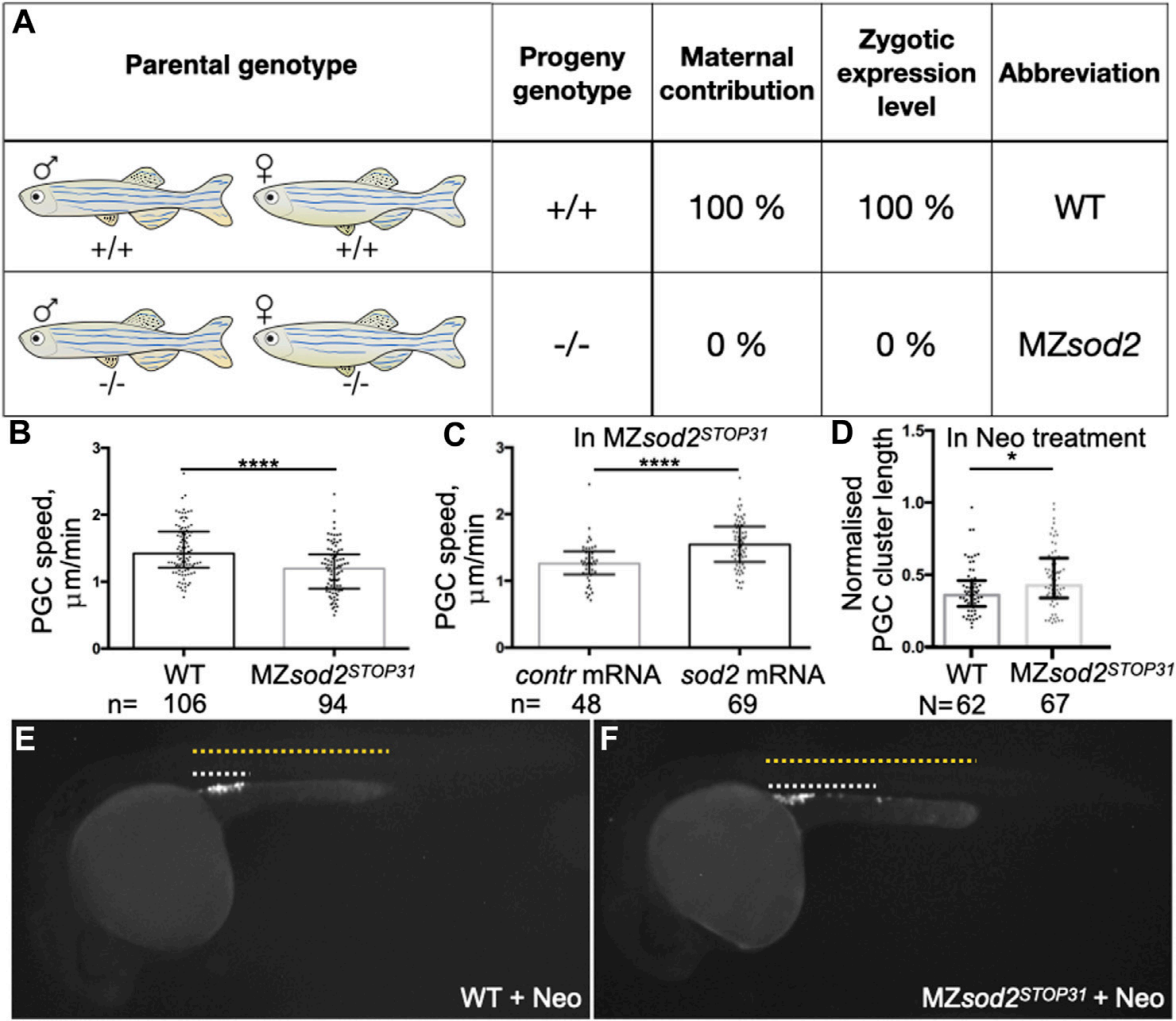
MZ*sod2* KO embryos exhibit reduction in the germ cell migration speed. **(A)** The scheme of crosses and genotypes analyzed in this manuscript. **(B, C)** PGC migration speed was determined by tracking cell migration for 70 min using Imaris software. **(D)** Normalized PGC cluster lengths at 24 hpf in WT and MZ*sod2* KO embryos treated with neomycin (enhanced ROS production condition). **(E, F)** Representative images of embryos analyzed for the PGC cluster length (quantification shown in **(D)**) ****p < 0.0001, *p < 0.05 was determined by Student’s t-test. n - number of PGCs analyzed in 38 WT and 36 MZ*sod2* KO embryos **(B)**, 24 MZ*sod2* KO + control (contr) mRNA and 29 MZ*sod2* KO + *sod2* mRNA embryos **(C)**. N - number of PGC clusters (one cluster per embryo) analyzed.

Interestingly, despite the central role that the protein plays in controlling ROS levels in the cell, under lab conditions the fish lacking the function of Sod2 were viable and developed into adults that proved to be fertile. This allowed us to generate and analyze embryos lacking both maternal and zygotically transcribed *sod2* RNAs (maternal-zygotic mutants (MZ), we designated as MZ*sod2* ^*STOP19*^ and MZ*sod2* ^*STOP31*^). The schemes of crosses and genotypes analyzed in this manuscript are presented in Figure 3A. In such embryos, presumably as a result of nonsense-mediated RNA decay, no *sod2* RNA (whole-mount *in situ* hybridization, Supplementary Figure S1B) or protein (Western blotting analysis, Figure 2B) could be detected.

Nevertheless, a phenotype we did observe in MZ mutant embryos at early somitogenesis stages (11 hpf) was that of a mild reduction in body size (Supplementary Figure S3A). However, as judged by RNA expression, patterning events progressed normally in all germ layers, i.e., ectoderm, mesoderm and endoderm (Supplementary Figure S3B, C), and the embryos appeared morphologically normal at 5 days of development. Relevant for this report, the expression of *cxcl12a*, namely, the chemokine guiding the migration of both pLLP and PGCs, was expressed normally in the mutant embryos (Supplementary Figure S3C).

### Sod2 function is dispensable for the collective migration of the pLLP cell cluster

The posterior lateral line primordium (pLLP) is a cluster of 100–150 cells that migrate collectively towards the tail of the zebrafish embryo (Haas and Gilmour, 2006). Interestingly, *sod2* mRNA was expressed within cells of the pLLP (Figure 1C, Supplementary Figure S1A), with elevated levels detected at the front of the cell cluster compared to the rear (Supplementary Figure S2). This expression pattern prompted us to examine the migration of the cell cluster in embryos lacking Sod2 function (MZ*sod2* ^*STOP19*^). As judged by the position of the pLLP at 24 hpf to 35 hpf (Supplementary Figure S4A), the cell clusters in mutant embryos migrated at the same speed as the non-manipulated wild-type clusters. Similarly, we did not observe an effect on the cluster size, suggesting a normal rate of cell division and death (S4B-B’’). Last, in mosaic pLLP clusters in which only some of the cells lacked Sod2 function and the rest were wild-type, we found no role of the protein in dictating the position of the cells within the primordium (Supplementary Figure S4C–E).

Thus, based on the parameters investigated in Supplementary Figure S4, Sod2 function was not essential for proper migration of the pLLP, nor for cell behavior within the cluster.

### Elevation of ROS affects PGC migration

**FIGURE 4:**
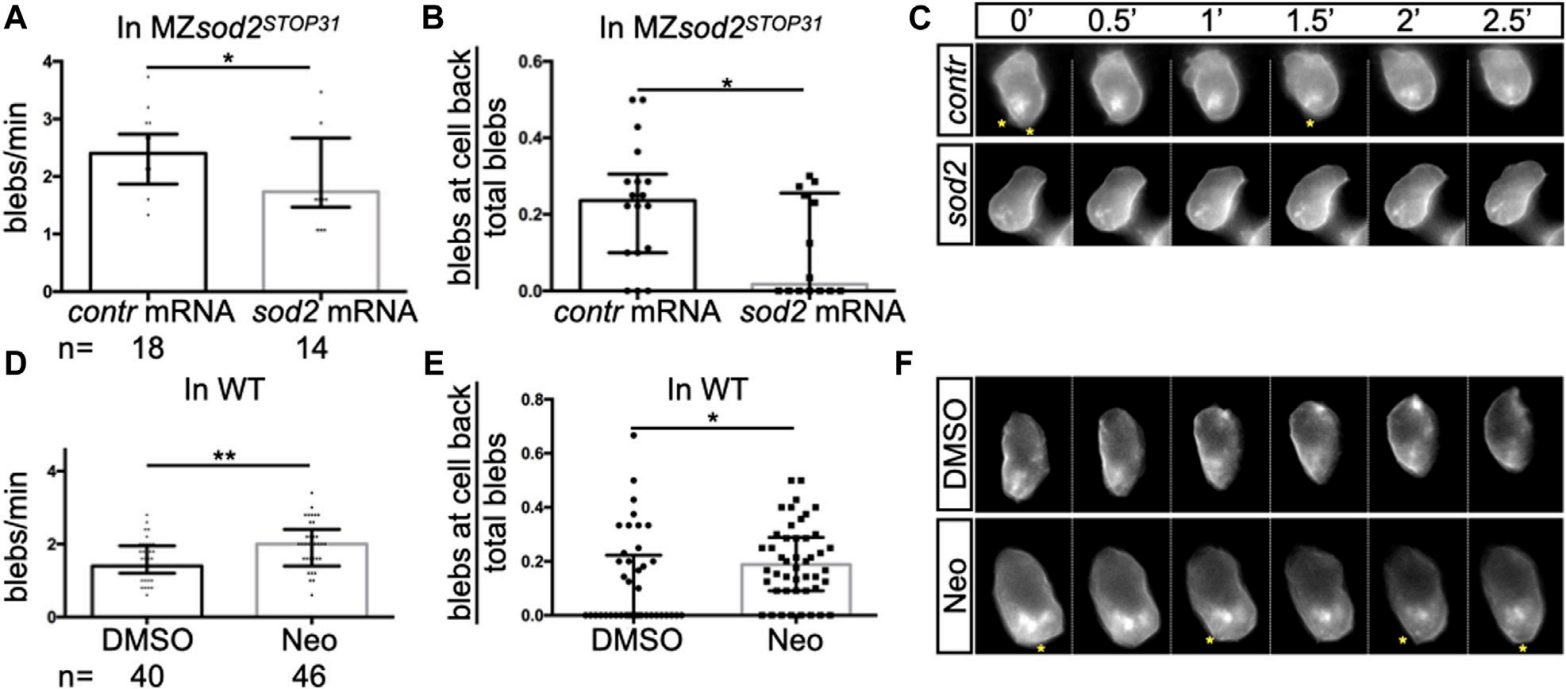
Defects in protrusion formation and germ cell polarity in *sod2* KO and neomycin-treated embryos. Analysis of **(A, D)** the number of blebs in PGCs per minute and **(B, E)** the proportion of blebs at the cell back. In graphs **A** and **B**, MZ*sod2* ^*STOP31*^ embryos were injected with control RNA or with RNA directing Sod2 expression to the germ cells (see Methods); the same cells were analyzed in panels **A** and **(B)**. In graphs **D** and **E**, wild-type embryos were treated with DMSO or with neomycin; the same cells were analyzed in panels **D** and **(E)**. **(C, F)** Snapshots from the representative time-lapse movies (movies S1 and S2) analyzed in **(A,B,D, E)**. Yellow asterisks point at blebs at the rear of cells. n - the number of PGCs analyzed. One PGC per embryo was imaged and analyzed. **p < 0.01, *p < 0.05, were calculated by Student’s t-test.

To investigate the possible function of Sod2 in another cell population that expresses the protein, we turned to primordial germ cells (PGCs). In different organisms PGCs are specified during early embryonic development and migrate as single cells from their site of specification to the region where the gonad develops to give rise to gametes, sperm and egg ((Richardson and Lehmann, 2010; Aalto et al., 2021; Grimaldi and Raz, 2020)). We examined the migration speed of PGCs in seven to nine hpf embryos and observed a mild reduction in the speed of *sod2* mutant PGCs as compared with their wild-type counterparts (Figure 3B). In line with this finding, cells devoid of Sod2 function were less polar as manifested by an increase in the rate of bleb-type protrusion formation, particularly at the cell rear (Figures 4A–C, Supplementary Movie S1). Conversely, overexpression of Sod2 led to an increase in migration speed, suggesting that the Sod2 activity level constitutes a limiting factor for germ cell translocation (Figure 3C). Despite the defects in protrusion formation and the slower migration of PGCs in MZ*sod2* ^*STOP31*^ embryos, normal size of PGC cluster formed at the region where the gonad develops (Supplementary Figure S5A).

In addition to the finding presented above, we detected higher ROS levels in cells deficient for Sod2 function (Supplementary Figure S5B). To examine whether Sod2 confers robustness to the migration process under conditions of increased oxidative stress in the PGCs, we subjected MZ*sod2* ^*STOP31*^ and wild-type embryos to neomycin (Neo), which was shown to increase oxidative stress in cells ((Harris et al., 2003; Esterberg et al., 2016)). Increasing the oxidative stress in this way led to an increase in bleb formation and a reduction in cell polarity (Figures 4D–F, Movie S2) in wild-type embryos. We next checked whether the function of Sod2 could confer robustness to the arrival of germ cells at their target under these conditions. Indeed, increasing ROS level by subjecting embryos to Neomycin further enhances the oxidative stress in cells lacking SOD2 function (Supplementary Figure S5C). Interestingly, loss of Sod2 function coupled to neomycin treatment affected PGC cluster formation at the region where the gonad develops (Figures 3D–F).

### Sod2 function is important for selection of fitter germ cells

**FIGURE 5:**
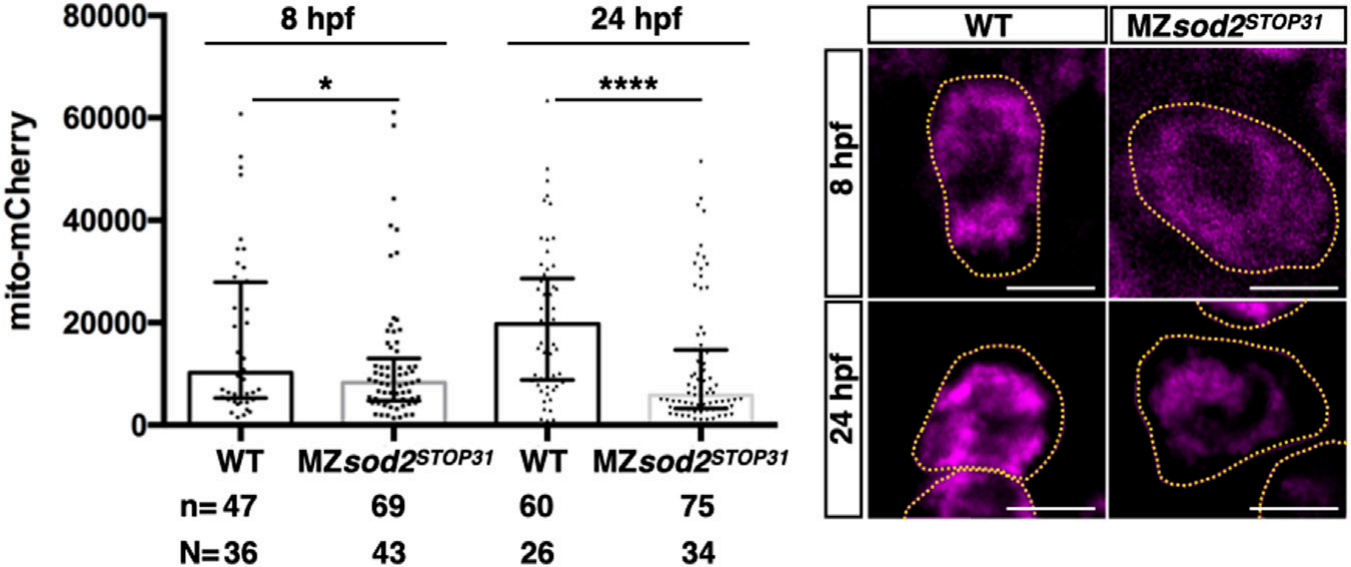
MZ*sod2* KO germ cells exhibit reduced mitochondria-derived fluorescence signal. Average intensity of the fluorescently-labeled mitochondria in WT and MZ*sod2* KO germ cells at 8 and 24 hpf. n - number of PGCs analyzed in N–number of embryos. ****p < 0.0001, *p < 0.05 was determined by ANOVA test. Scale bar 10 µm.

To further examine the function of Sod2 in the germline, we monitored the mitochondria, the organelle within which the enzyme functions. Measuring the fluorescence intensity levels of a mitochondria-directed mCherry protein in 8 and 24 hpf embryos revealed a very strong reduction in the average fluorescence levels particularly in MZ*sod2* ^*STOP31*^ PGCs at 24 hpf as compared with their wild-type counterparts (Figure 5, Supplementary Figure S6).

It has previously been reported that, as quantified by the amount of mitochondrial DNA, the number of mitochondria decreases exponentially from the time of egg fertilization until late larval stages, when replication of these organelles reinitiates (Otten et al., 2016). In light of this, we hypothesized that the further decrease in mitochondria-derived fluorescence signal in MZ*sod2* ^*STOP*^ germ cells could result in reduced fitness of these cells relative to wild-type cells during later stages of development (Figure 9).

**FIGURE 6:**
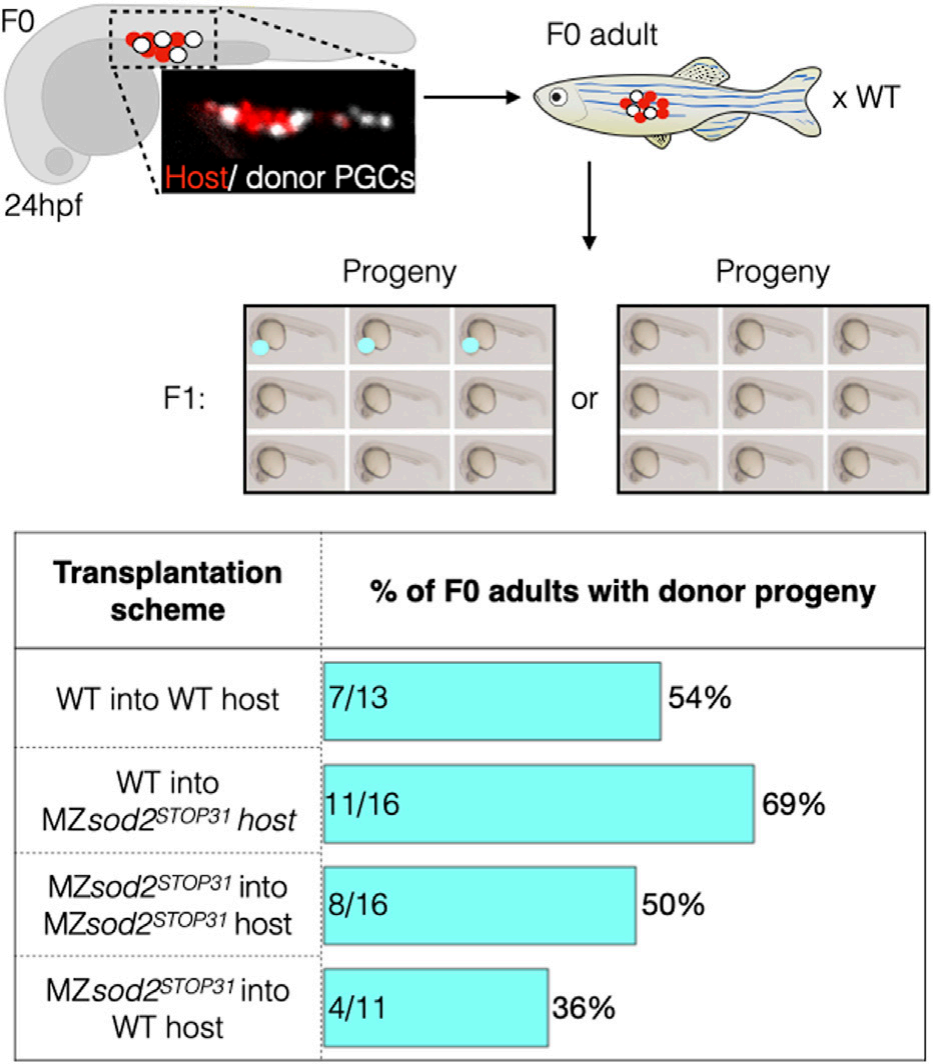
Analysis of zebrafish with mosaic gonads: PGC transplantation approach. Embryos with mosaic germ cell clusters (white cells are the transplanted donor PGCs, red cells are the host PGCs in the upper panels) were selected at 24 hpf and raised to adulthood and crossed to wild-type fish. If clutches contained embryos with the dominant marker of the donor line (red eyes), the outcrossed adult fish would be counted as “positive.” Lower graph shows the number of such positive fish relative to the total number of adults tested is presented in percentage for each background. Numbers within the bars represent the number of F0 adults with donor progeny divided by the total number of F0 adults raised for this line.

To test this hypothesis, we transplanted germ cells from wild-type and MZ*sod2* ^*STOP31*^ lines into either wild-type or MZ*sod2* ^*STOP31*^ hosts, with the transplanted donor germ cells carrying a dominant marker (fluorescent eyes, Supplementary Figure S7). Chimeric embryos containing both host and donor germ cells were raised and mated with wild-type fish, and we scored for the embryos expressing the donor dominant marker (fluorescent eyes), which signifies the fitness of transplanted PGCs (Figure 6).

Following this experimental scheme, we detected F1 progeny derived from the transplanted germ cells in 7 of 13 (54%) adult fish when wild-type donor PGCs were transplanted into wild-type hosts. Similarly, 8 out of 16 (50%) adult fish had progeny expressing the donor domain marker when MZ*sod2* ^*STOP31*^ PGCs were transplanted into MZ*sod2* ^*STOP31*^ hosts. The result of the experiment changed when germ cells were transplanted into heterologous backgrounds. In these cases, F1 progeny expressing donor dominant makers were observed in 11 of 16 (69%) animals in which wild-type germ cells transplanted into the MZ*sod2* ^*STOP31*^ background. Conversely, the number of adult wild-type hosts giving rise to dominant-marker carrying progeny was only 4 of 11 fish (36%) in the case of MZ*sod2* ^*STOP31*^ cells transplanted into wild-type hosts.

**FIGURE 7:**
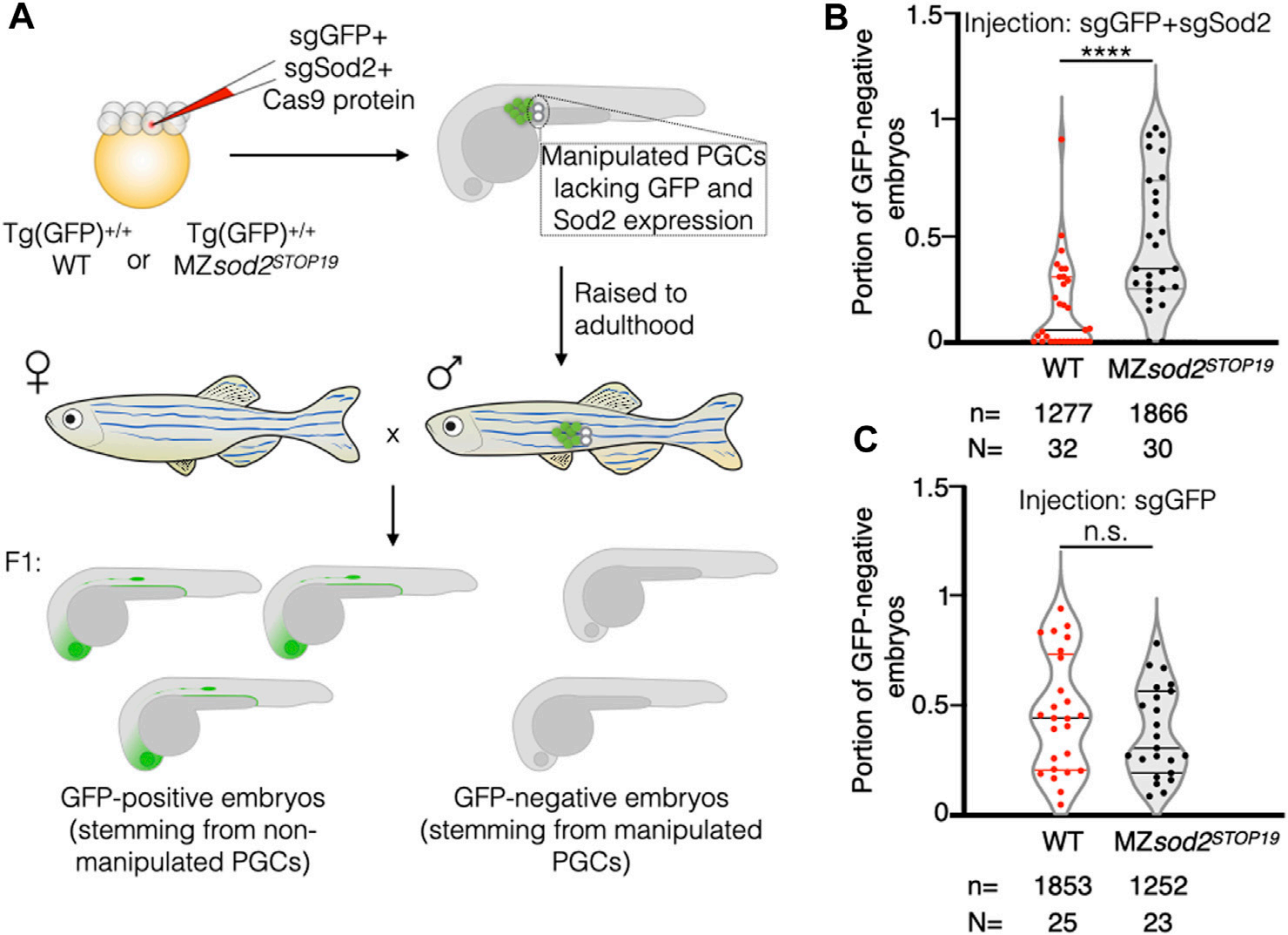
Generation and analysis of zebrafish with mosaic gonads: Cas9-based approach. **(A)** Embryos of WT or *sod2* KO backgrounds homozygous for *Tg(cldnB:lynEGFP)* (*Tg(GFP)* ^*+/+*^) were injected at the 8-cell stage into one of the middle blastomeres with Cas9 protein and two guide RNAs targeting *egfp* and *sod2* sequences (sgEGFP and sgSod2). This manipulation affected only a fraction of germ cells per embryo, allowing for a high-throughput generation of animals with mosaic gonads containing WT germ cells (expressing Sod2 and EGFP transgene) and KO germs cells (where both Sod2 and EGFP functions were eliminated). Such embryos were raised to adulthood. Adult males were outcrossed to WT females, and the percentage of GFP-negative embryos (lacking EGFP and Sod2 expression) within the clutch was calculated. Results are presented in graph **(B)**. The results of the negative control experiment with animals injected only with sgGFP (manipulating GFP expression only) are presented in graph **(C)**. n–number of embryos and N - number of adult fish analyzed. ****p < 0.0001, ^n.s.^ *p* > 0.05 was determined by Student’s t-test.

Due to the complexity and low throughput of the experiment presented above, only a relatively small number of chimeric animals could be generated. Thus, as an additional test for the hypothesis, we generated fish with chimeric gonads containing wild-type and MZ*sod2* ^*STOP19*^ germ cells using a different approach (Figure 7A). To this end, we co-injected Cas9 protein and guide RNAs targeting *egfp* and *sod2* sequences into 1 blastomere of 8-cell-stage wild-type and MZ*sod2* ^*STOP19*^ embryos. The injected embryos were homozygous for an EGFP transgene. The injected embryos were raised, and adult males were crossed with wild-type female fish, with the progeny analyzed for the percentage of embryos lacking a GFP signal. Thus, knocking out *sod2* according to this scheme resulted in adult fish with a chimeric germline, where progeny lacking the EGFP signal representing the proportion of *sod2* KO germ cells that contributed to the germline. Intriguingly, we found that the potency of cells lacking Sod2 to contribute to the germline and form gametes was very strongly reduced in wild-type animals as compared to their ability to do so when located within a gonad containing only Sod2 mutant cells (Figure 7B). We thus conclude that while Sod2 mutant cells can generate gametes, they are much less likely to do so when positioned within an environment containing wild-type cells. Thus, when located within an environment of wild-type cells, the Sod2-deficient germline cells are outcompeted and contribute less to the gametes (WT in Figure 7B). Importantly, the effect of germ cell competition was not observed in similarly generated control chimeric gonads of embryos injected only with guide RNA for the *egfp* sequence (no *sod2* knockout) (Figure 7C). Together, Sod2 deficient cells contribute less to the germline when located among wild-type germ cells. In contrast, the negative controls where Sod2 activity was not altered (anti *gfp* guides, or anti *sod2* guides in *sod2* mutant cells) the treated cells showed no difference in their contribution to the germline as compared with non-treated cells. Consistent with the importance of reduced ROS levels in germline cells for proper gametogenesis, MZ*sod2* homozygous mutant fish are less fertile (Supplementary Figure S8).

**FIGURE 8:**
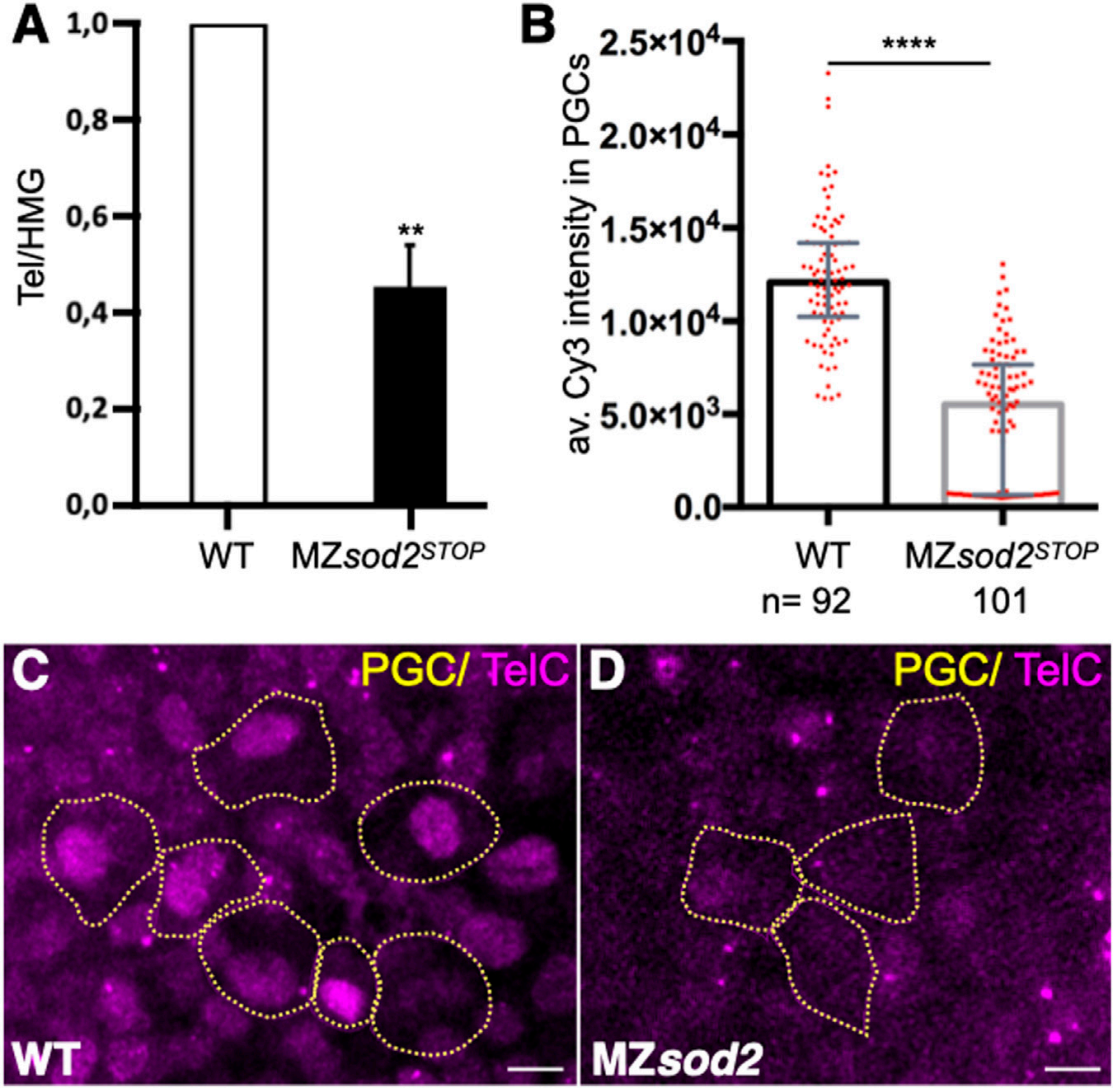
The number of telomeric repeats is reduced in *sod2* KO fish lines. **(A)** The relative length of telomeric repeats in wild type and MZ*sod*2 mutants was determined by qPCR (graph). The qPCR values were normalized to the WT condition. **(B)** The relative number of telomeric repeats was determined by fluorescence *in situ* hybridization (FISH) using Cy3-conjugated TelC probes. The graph presents intensity measurements of the TelC signal in individual PGC nuclei. n–number of PGCs analyzed in 32 WT and 35 MZ*sod2* embryos **(C, D)** Representative confocal images of TelC-FISH staining. PGCs are identified by the expression of EGFP-F′ on their cell membranes (represented by yellow dotted lines). ****p < 0.0001was determined by Student’s t-test. Scale bar 10 µm.

An interesting phenotype we observed, which may be relevant for the reduced potential of Sod2-depleted germ cells to generate gametes as compared to non-manipulated neighboring germ cells, is a reduction in the number of telomeric repeats in MZ*sod2* KO embryos. This analysis was performed using qPCR (Figure 8A), or fluorescence *in situ* hybridization using the telomere-specific probe (TelC-Cy3, Figures 8B–D, Supplementary Figure S9). A possible basis for the shortening of telomeres is DNA damage that telomere length reports on. The reduction in telomere length we observe in *sod2* mutants could affect the fitness of cells that carry the genetic information to future generations.

## Discussion

The Sod2 protein has been shown to be involved in a range of physiological and pathological processes. For example, the *Drosophila sod2* mutant (*SOD2* ^*Bewildered*^) exhibits aberrant brain morphology, abnormal axonal targeting, and neurodegeneration (Celotto et al., 2012). We observed strong expression of *sod2* mRNA in the zebrafish brain as well, but, based on the molecular markers we examined and the apparent normal behavior of MZ*sod2* adult fish, the loss of Sod2 in zebrafish did not result in a pronounced phenotype in this tissue. Since the ROS level has been shown to affect lipid metabolism ((Quijano et al., 2016; Zhuang et al., 2021)), the main energy source of the early embryo, the reduction in body size of 12-hour-old MZ*sod2* ^*STOP*^ mutant embryos could stem from defects in the availability of these molecules (reviewed in (Sant and Timme-Laragy, 2018; Quinlivan and Farber, 2017)).

The PGC migration phenotype we observed in MZ*sod2* mutants and the similar phenotype of neomycin-treated embryos could reflect defects in energy production as well, which in this case could stem from defects in the function of the mitochondria (Esterberg et al., 2016). We attribute the lack of migration defects in the pLLP to the more robust nature of collective cell migration in which mild locomotion aberrations in some cells can be compensated by other cells in the cluster ((Knutsdottir et al., 2017; Yamaguchi et al., 2022)).

In contrast to the very mild or lack of phenotypes we observed in PGCs during early stages of embryonic development (first 1–2 days post fertilization), the phenotypes we detected at later stages were much more pronounced. A possible explanation could be that the PGC competition phenotype we observe reflects a cumulative effect of lack of Sod2 function over the 3-month period between the establishment of the germline and the generation of gametes.

A frequently documented type of cellular heterogeneity that is subjected to selection has been described in somatic cells in the form of mitochondrial heteroplasmy (e.g., mitochondrial function in human cells (Wei et al., 2019)). In this case, cells with an elevated proportion of defective mitochondria are eliminated by more robust neighboring cells in a process termed “purifying selection” (Lima et al., 2021). Heterogeneities in cellular functions have also been identified in other cell populations including stem cells ((Ellis et al., 2019; Lawlor et al., 2020; Lima et al., 2021), reviewed in (Muller-Sieburg et al., 2012; Krieger and Simons, 2015)). In germ cells, such heterogeneities can be especially consequential, as they are transmitted to the next-generation (Zhang et al., 2022).

**FIGURE 9:**
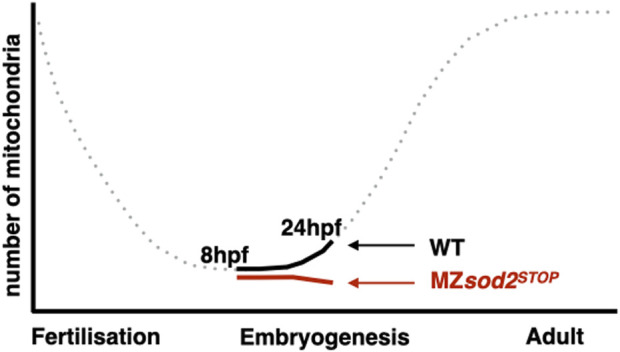
Schematic illustration of changes in mitochondria numbers during zebrafish development. Representation of mitochondria numbers per germ cell at 8 hpf and 24 hpf of zebrafish development in WT (black) and MZ*sod2* KO (red) animals. The dotted part of the graph is based on (33).

Since during the embryonic stages studied here there was no *de novo* production of mitochondria, the number of the maternally provided organelles per cell declined with each cell division, reaching a very low number (estimated to be 100 per cell (Otten et al., 2016)). Indeed, the time of PGC migration we examined here corresponds to developmental stages with very low numbers of mitochondria per cell (Otten et al., 2016). The small number of mitochondria is an obvious parameter relevant for mitochondrial heteroplasmy in PGCs and for the so called mitochondrial “bottleneck” effect (Otten et al., 2016). In the case of germ cells, this time point can serve as the stage when purifying selection takes place, contributing to the transmission of healthier mitochondria to the progeny. Consistent with the Sod2 level being important for mitochondria function and DNA integrity, in MZ*sod2* ^*STOP*^ embryos the already low mitochondria number was further reduced (schematically presented in Figure 9), and telomeres were shorter. The low level of mitochondria observed in PGCs in MZ*sod2* ^*STOP*^ embryos and the aberrations in migration motivated us to study the possible evolutionary role of Sod2 in PGC maintenance and natural selection. Indeed, the results of the germline transplantation experiments (Figure 6) and those of the chimeric germline analysis (Figure 7) show that germ cells lacking Sod2 were less likely to generate gametes as compared with wild-type PGCs when positioned together within the developing gonad. Since such phenomenon has not been observed among cells of similar genotypes, these results fit the definition of cell competition ((de la Cova et al., 2004; Moreno and Basler, 2004)), which in this case involves germline cells. In the context of this work, such competition could select for germline cells with lower ROS-generated damage, thereby controlling the quality of gametes and, thereby, the fitness of somatic cells in the next-generation. Thus, it is likely that the full manifestation of the MZ*sod2* ^*STOP*^ phenotype may only be appreciated on the evolutionary scale when followed over several generations.

## Data Availability

The raw data supporting the conclusions of this article will be made available by the authors, without undue reservation.
