# Supplementary material for: The mitochondrial protein Sod2 is important for the migration, maintenance, and fitness of germ cells
Source: Front Cell Dev Biol. 2023 Oct 26;11:1250643. doi: 10.3389/fcell.2023.1250643 (PMC10639133; doi:10.3389/fcell.2023.1250643)
Supplement: Supplementary file 2 [file Table1.docx]

**Supplementary Figures and Captions**

**The mitochondrial protein Sod2 is important for the migration, maintenance, and fitness of germ cells**

Katsiaryna Tarbashevich^1^, Laura Ermlich^1^, Julian Wegner^1^, Jana Pfeiffer^1^ and Erez Raz^1,2,*^

^1^ Institute of Cell Biology, ZMBE, Von-Esmarch-Str. 56, 48149 Muenster, Germany

^2^ Max Planck Institute for Molecular Biomedicine, D-48149, Münster, Germany.

*correspondence

Prof. Erez Raz

(erez.raz@uni-muenster.de)

**Supplementary Figures**

**Fig S1. RNAscope-based quantification of the *sod2* expression domains in the wild-type embryos.** (A) *sod2* expression was calculated as a ratio of *sod2* positive domain (pLLP, for example, yellow dotted outline in lower panels) to the nearby *sod2* negative region (white dotted outline in the lower right panel) of the same area on single-slice confocal images using Fiji software. The panels are the representative images illustrating the principle of calculation. *sod2* mRNA is presented in yellow, *cldnB* expression was used to mark the migrating pLLP (green). N- number of embryos analyzed, n – number of expression domains analyzed. Error bars depict SEM. Scale bar 15 µm. (B) *sod2* mRNA expression in MZ*sod2^STOP^* embryos at 24hpf.

**Fig S2. RNAscope analysis of the *sod2* expression in the pLLP and germ cells at 24 hpf.** *sod2* mRNA is presented in red. pLLP is marked by the expression of *cldnB* (green, lower left panels), germ cells are marked by the expression of the germline marker *vasa* RNA (magenta, lower right panels). The white arrow depicts the direction of pLLP migration.


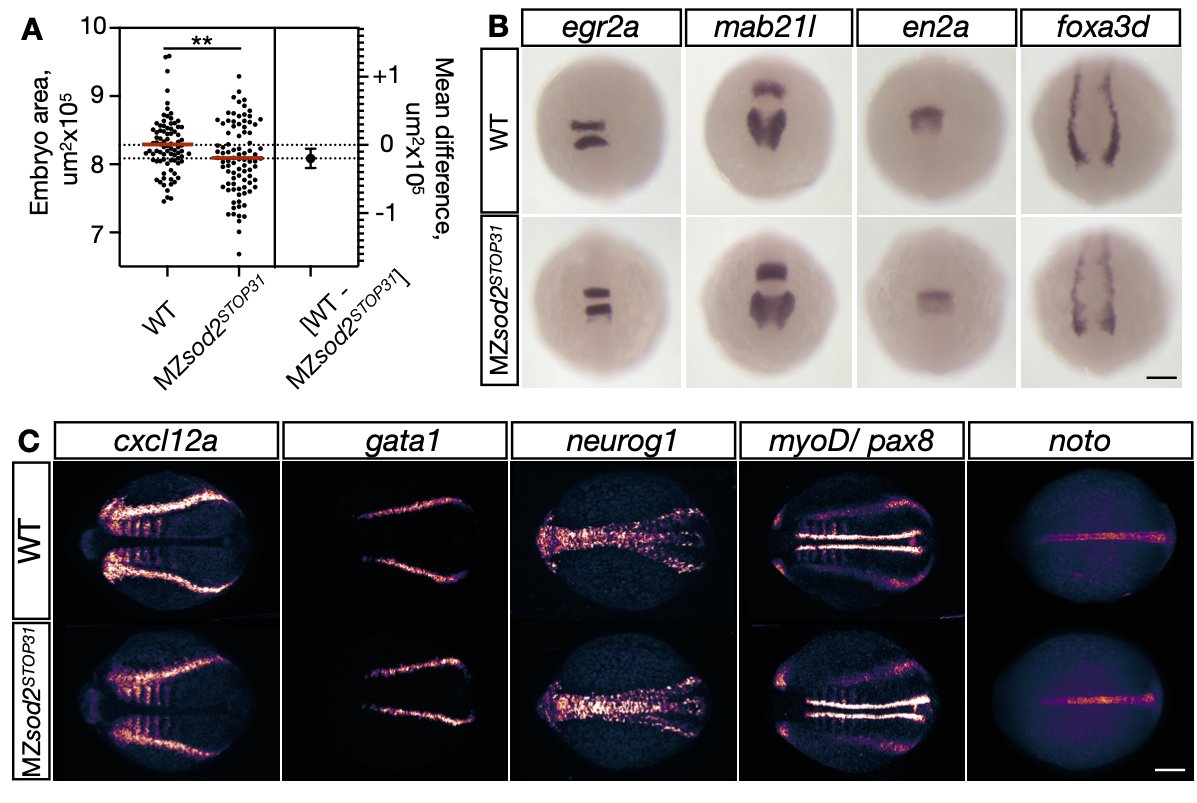


**Fig S3. General embryonic patterning is not affected in *sod2* KO lines. (A)** Analysis of the size of wild-type (WT) and *sod2*-KO (MZ*sod2^STOP31^*) 11 hpf embryos. **(B)** Expression patterns of *egr2a*, *mab21l*, *en2a­,* and *foxa3d* determined by whole-mount in situ hybridization for WT (upper panels) and MZ*sod2^STOP31^* (lower panels) embryos. **(C)** Expression patterns of *cxcl12a*, *gata1*, *neurog1, myoD, pax2,* and *noto* determined by RNAscope for WT (upper panels) and MZ*sod2^STOP31^* (lower panels) embryos. **P<0.01 was determined by Student’s t-test. Scale bars 100 µm. Error bars depict SEM.

**Fig S4. Sod2 function is not essential for proper migration and size of the pLLP. (A)** The analysis of normalized migration distance of individual pLLPs in 37 WT and of 34 MZ*sod2* KO embryos at 35 hpf. (B-B’’) Graph **(B)** represents the analysis of normalized volumes of individual pLLPs of 44 WT and of 44 MZ*sod2* KO embryos at 35 hpf. **(B’)** A representative image of a migrating pLLP used for the Imaris-based surface rendering and **(B’’)** volume determination. Scale bar 20 µm. **(C)** Snapshots from an 8-hour time-lapse-based analysis of the positioning of transplanted MZ*sod2* KO pLLP cells (red) into a WT cell cluster (28 to 36 hpf). Scale bar 40 µm. **(D-D’’)** Representative maximum intensity projection images of three pLLPs after co-transplantation experiments of WT (blue) and *sod2* KO (red) cells into WT hosts to analyze cell positioning and survival within the migrating pLLPs at 35 hpf. Scale bar 50 µm. **(E)** Quantification of the positioning of transplanted WT and *sod2* KO cells within WT pLLPs. The percentage was determined relative to the total number of transplanted cells for a given cohort (WT or *sod2* KO cells). Three independent experiments were summarized and 68 pLLPs were analyzed; error bars represent the standard deviation (SD). No significant difference between WT or *sod2* KO groups was detected by Student’s t-test (P>0.05). Error bars depict SEM.

**Fig S5. Elevated ROS level does not affect PGC arrival and clustering at the gonad region at 24hpf. (A)** Normalized PGC cluster lengths at 24 hpf of WT and MZ*sod2* KO embryos. N - number of PGC clusters (embryos) analyzed. **(B)** The graph illustrates the level of the oxidative stress in WT and MZ*sod2* KO cells quantified by the average fluorescence (mean grey value) of CellRox on single-slice confocal images using Fiji software. Panels to the right are representative examples of images used for the quantification. **(C)** The graph illustrates the level of the oxidative stress in MZ*sod2* KO treated with DMSO (control condition) or Neomycin. The level of ROS was quantified by the average fluorescence (mean grey value) of CellRox on single-Z-plane confocal images using Fiji software. Panels to the right are representative examples of images used for the quantification. N -number of embryos analyzed, n – number of cells analyzed.

Statistical significance was determined by Student’s t-test: ****P<0.0001, ***P<0.001, ^n.s.^P>0.05. Error bars depict SEM. Scale bar 10 µm.

**Fig S6. MZ*sod2* KO germ cells of both KO lines generated exhibit reduced mitochondria-derived fluorescence signal** **at 24 hpf.** Average intensity of the fluorescently labeled mitochondria in WT and MZ*sod2* KO germ cells. The intensity was measured in the incross of MZ*sod2STOP^31^* mutants and in the intercross MZ*sod2^STOP31^* x MZ*sod2^STOP19^* mutants. Representative images (panels below the graph) are scaled for the fluorescence relative to the WT condition. Yellow dotted line depicts the PGC membrane. n = number of PGCs and N – number of embryos analyzed. ****P<0.0001 was determined by ANOVA test. Error bars depict SEM. Scale bar 10 µm.

**Fig S7.** **Generation of zebrafish with mosaic gonads: PGC transplantation approach.** WT or *sod2* KO germ cells from donor embryos expressing a farnesylated EGFP (EGFP-F’) transgene (white PGCs) and the dominant marker (fluorescent eyes) were transplanted into hosts expressing an mCherry-F’ transgene (red PGCs). Embryos with mosaic germ cell clusters were selected at 24 hpf and raised to adulthood.

**Fig S8. Percentage of WT and MZ*sod2* crosses laying eggs.** Percentage of 1- and 2-years old wild-type and MZ*sod2* fish crosses laying eggs was calculated for 38 couples for each condition and averaged for three independent experiments. Error bars represent SEM.

**Fig S9. TelC-Cy3 and TelC-Cy5 immunostaining in the WT and MZ*sod2^STOP^* embryos at 12hpf.** In the panels are presented confocal images scaled for the fluorescence relative to the WT condition. Negative controls were not hybridized with TelC probes. Scale bar 10 µm.

**Movie S1.** Time-lapse analysis of PGC migration in MZ*sod2^STOP^* embryos injected with control mRNA or *sod2* mRNA (rescue of the phenotype). The front of the cells is pointing upwards. In the control movie (right), less focused blebbing is observed, with blebbing activity seen at the cell back (bottom). This phenotype is reversed by injection of Sod2-encoding RNA (left movie). Yellow asterisks point at blebs at the rear of cells.

**Movie S2.** Time-lapse analysis of PGC migration in wild-type embryos treated with DMSO (control condition, left) or 400uM neomycin (right movie). PGCs treated with neomycin are less polar and form blebs also in the cell back (bottom). Yellow asterisks point at blebs at the rear of cells.
